# Diammonium aqua­(ethyl­ene­diamine­tetra­acetato)iron(II) trihydrate

**DOI:** 10.1107/S1600536808042190

**Published:** 2008-12-17

**Authors:** Hua-Ming Huang, Hong-Bing Yang, Xiang-Yu Li, Fang-Fang Ren

**Affiliations:** a College of Chemistry and Chemical Engineering, Luoyang Normal University, Luoyang 471022, People’s Republic of China

## Abstract

In the title compound, (NH_4_)_2_[Fe(C_10_H_12_N_2_O_8_)(H_2_O)]·3H_2_O, the Fe^II^ center is in a distorted penta­gonal-bipyramidal geometry. Two carboxyl­ate O and two N atoms from the ethyl­enediaminetetra­acetate (EDTA) ion and one O atom from coordinated water comprise the equatorial plane. Two other carboxyl­ate O atoms from the EDTA ion occupy the apical sites. Both ammonium cations and all water mol­ecules function as hydrogen-bond donors, and ten N—H⋯O and nine O—H⋯O hydrogen bonds form a three-dimensional network between the complex anions, cations and the water mol­ecules.

## Related literature

For an eight-coordinate Eu(II)–EDTA polymer complex, see: Janicki *et al.* (2005 ▶). For seven-coordinate EDTA-aqua vanadate(III) complexes, see: Shimoi *et al.* (1991 ▶). For hydrate structures of [Fe(III)EDTA(H_2_O)]^−^ anions with Na, Ag, K, and Tl cations, see: Solans *et al.* (1984 ▶). For high-concentration EDTA ferric ammonium salts solution, applied in the photographic processing of films and paper, see: Wang *et al.* (1999 ▶). For the preparative method of high-concentration ferric ammonium ethyl­ene diamine tetra­acetate solution, see: Zheng *et al.* (2006 ▶). 
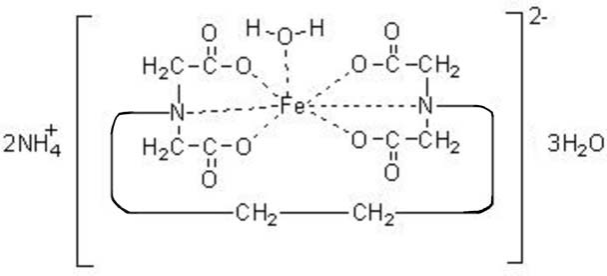


## Experimental

### 

#### Crystal data

(NH_4_)_2_[Fe(C_10_H_12_N_2_O_8_)(H_2_O)]·3H_2_O
*M* *_r_* = 452.21
Triclinic, 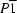

*a* = 8.7615 (10) Å
*b* = 8.9485 (10) Å
*c* = 13.4742 (15) Å
α = 80.3090 (10)°
β = 81.0050 (10)°
γ = 68.7790 (10)°
*V* = 965.47 (19) Å^3^
*Z* = 2
Mo *K*α radiation
μ = 0.85 mm^−1^
*T* = 291 (2) K
0.49 × 0.43 × 0.36 mm

#### Data collection

Bruker SMART CCD area-detector diffractometer
Absorption correction: multi-scan (*SADABS*; Bruker, 1997 ▶) *T*_min_ = 0.682, *T*_max_ = 0.748
7220 measured reflections
3455 independent reflections
3162 reflections with *I* > 2σ(*I*)
*R*_int_ = 0.014

#### Refinement

*R*[*F* ^2^ > 2σ(*F* ^2^)] = 0.029
*wR*(*F* ^2^) = 0.077
*S* = 1.06
3455 reflections
244 parameters
H-atom parameters constrained
Δρ_max_ = 0.64 e Å^−3^
Δρ_min_ = −0.42 e Å^−3^

### 

Data collection: *SMART* (Bruker, 1997 ▶); cell refinement: *SAINT* (Bruker, 1997 ▶); data reduction: *SAINT*; program(s) used to solve structure: *SHELXS97* (Sheldrick, 2008 ▶); program(s) used to refine structure: *SHELXL97* (Sheldrick, 2008 ▶); molecular graphics: *SHELXTL* (Sheldrick, 2008 ▶); software used to prepare material for publication: *SHELXTL*.

## Supplementary Material

Crystal structure: contains datablocks global, I. DOI: 10.1107/S1600536808042190/si2133sup1.cif

Structure factors: contains datablocks I. DOI: 10.1107/S1600536808042190/si2133Isup2.hkl

Additional supplementary materials:  crystallographic information; 3D view; checkCIF report

## Figures and Tables

**Table 1:** Selected bond lengths (Å)

Fe1—O5	2.1362 (15)
Fe1—O7	2.1736 (13)
Fe1—O3	2.2036 (15)
Fe1—O1	2.2603 (13)
Fe1—O9	2.2741 (14)
Fe1—N1	2.3015 (16)
Fe1—N2	2.3087 (15)

**Table 2:** Hydrogen-bond geometry (Å, °)

*D*—H⋯*A*	*D*—H	H⋯*A*	*D*⋯*A*	*D*—H⋯*A*
N4—H4*F*⋯O2^i^	0.92	1.93	2.821 (2)	166
N4—H4*E*⋯O7^ii^	0.86	2.54	2.990 (2)	114
N4—H4*E*⋯O7^iii^	0.86	2.15	2.924 (2)	149
N4—H4*D*⋯O3^ii^	0.82	2.04	2.844 (2)	167
N4—H4*C*⋯O4^iv^	0.80	2.14	2.928 (2)	171
N3—H3*D*⋯O4^ii^	0.82	2.11	2.919 (2)	172
N3—H3*C*⋯O11	0.83	2.29	3.083 (3)	159
N3—H3*B*⋯O9^i^	0.84	2.60	3.184 (3)	127
N3—H3*B*⋯O1^i^	0.84	2.29	3.121 (2)	167
N3—H3*A*⋯O4	0.86	2.22	3.081 (3)	174
O12—H8*W*⋯O6^i^	0.84	1.95	2.783 (2)	170
O12—H7*W*⋯O10^v^	0.83	1.92	2.732 (3)	167
O11—H6*W*⋯O12^vi^	0.83	1.93	2.749 (2)	169
O11—H5*W*⋯O5^i^	0.83	2.66	3.213 (2)	125
O11—H5*W*⋯O6^i^	0.83	1.94	2.750 (2)	165
O10—H4*W*⋯O2^i^	0.85	1.92	2.767 (3)	179
O10—H3*W*⋯O11	0.85	1.88	2.727 (3)	179
O9—H2*W*⋯O8^vii^	0.84	1.96	2.753 (2)	159
O9—H1*W*⋯O1^viii^	0.83	2.11	2.920 (2)	166

Symmetry codes: (i) 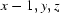; (ii) 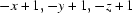; (iii) 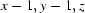; (iv) 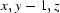; (v) 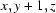; (vi) 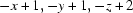; (vii) 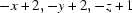; (viii) 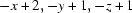.

**Fig. 1.**
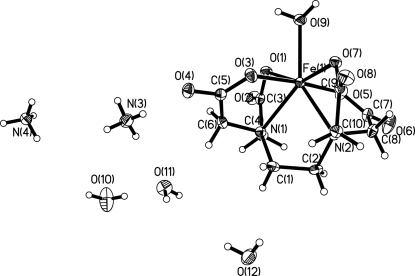
The molecular structure of the title compound with atom labelling of non-hydrogen atoms. Displacement ellipsoids are drawn at the 30% probability level.

**Fig. 2.**
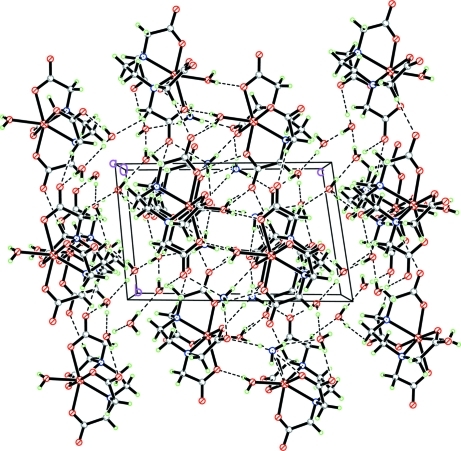
The Packing diagram for the title compound.

## supplementary crystallographic information

### Comment

High concentrated EDTA ferric ammonium salts solution is an important oxidizer and bleacher in the photographic processing of film or paper because it can oxidize metallic silver in the film into silver ions that can be dissolved in the solution (Wang *et al.* 1999) and (Zheng *et al.* 2006). In the present study, we try to improve the production method by H4EDTA and ammonia reacted with ferric oxide.

In the title compound the iron atom adopts a distorted pentagonal bipyramidal geometry with a hexadentate EDTA ligand and an aqua ligand. The molecular structure unit (Fig.1) consists of one iron(II) ion, one ethylenediamine tetraacetic acid ion, two ammonium ions, one coordinated water molecule and three lattice water molecules. Two carboxylate oxygen atoms and two nitrogen atoms from the EDTA ion and one oxygen atom from the coordinated water comprise the equatorial basal plane, and two other carboxylate oxygen atoms from the EDTA ion occupy the apical sites in the title structure (Fig. 1). The Fe–O and Fe–N bond distances are in the range of 2.1362 (15) - 2.2741 (14) Å and 2.3015 (16) - 2.3087 (15) Å, respectively (Table 1).

The title  structure is quite similar to those of [Fe(III)(EDTA)(H_2_O)]^-^ , although the molecules have different cations (Na, Ag in a six-coordinate, and K and Tl in a seven coordinate fashion to oxygen atoms of water and C==O groups of EDTA), and the iron(III) atoms exhibit different distorted pentagonal bipyramidal coordination geometries (Solans *et al.* 1984). Similar coordination spheres were found in seven-coordinate EDTA-aqua-vanadate(III) complexes, and they were described as capped trigonal prisms (Shimoi *et al.* 1991). An eight-coordinate polymeric Eu(II)EDTA chain structure with guanidine cations and crystal water was discussed by Janicki *et al.* (2005), and the coordination figure described as a distorted dodecahedron.

In the title compound, there are many ammonium cations and water molecules between the complex anions (Fig. 2). Both ammonium cations and all water molecules function as hydrogen bond donors. Ten intermolecular N—H···O and nine O—H···O hydrogen bonds form a three-dimensional network (Table 2).

### Experimental

The title compound was prepared by H4EDTA reacted with ferric oxide and ammonia in a water solution at 365 K over 3 hours, and recrystallized from water solution at room temperature yielding colorless crystals suitable for single-crystal X-ray diffraction. In the experimental process, it was found that a large number of precipitates were formed.

### Refinement

The positions of water H and ammonium H atoms were located in a difference Fourier map and were allowed to ride with distances between 0.82 Å and 0.92 Å and with *U*_iso_(H) = 1.5 *U*_eq_(O,N). Methylene H atoms were positioned geometrically and refined as riding atoms, with C-H = 0.97 Å (CH2) and *U*_iso_(H) = 1.2Ueq(C).

### Crystal data

**Table:**

(NH_4_)_2_[Fe(C_10_H_12_N_2_O_8_)(H_2_O)]·3H_2_O	*Z* = 2
*M**_r_* = 452.21	*F*(000) = 476
Triclinic, *P*1	*D*_x_ = 1.556 Mg m^−^^3^
*a* = 8.7615 (10) Å	Mo *K*α radiation, λ = 0.71073 Å
*b* = 8.9485 (10) Å	Cell parameters from 4241 reflections
*c* = 13.4742 (15) Å	θ = 2.5–28.2°
α = 80.309 (1)°	µ = 0.85 mm^−^^1^
β = 81.005 (1)°	*T* = 291 K
γ = 68.779 (1)°	Block, colorless
*V* = 965.47 (19) Å^3^	0.49 × 0.43 × 0.36 mm

### Data collection

**Table:**

Bruker SMART CCD area-detector diffractometer	3455 independent reflections
Radiation source: fine-focus sealed tube	3162 reflections with *I* > 2σ(*I*)
graphite	*R*_int_ = 0.014
φ and ω scans	θ_max_ = 25.5°, θ_min_ = 2.5°
Absorption correction: multi-scan (*SADABS*; Bruker, 1997)	*h* = −10→7
*T*_min_ = 0.682, *T*_max_ = 0.748	*k* = −10→10
7220 measured reflections	*l* = −16→16

### Refinement

**Table:**

Refinement on *F*^2^	Primary atom site location: structure-invariant direct methods
Least-squares matrix: full	Secondary atom site location: difference Fourier map
*R*[*F*^2^ > 2σ(*F*^2^)] = 0.029	Hydrogen site location: inferred from neighbouring sites
*wR*(*F*^2^) = 0.077	H-atom parameters constrained
*S* = 1.06	*w* = 1/[σ^2^(*F*_o_^2^) + (0.0388*P*)^2^ + 0.4698*P*] where *P* = (*F*_o_^2^ + 2*F*_c_^2^)/3
3455 reflections	(Δ/σ)_max_ = 0.001
244 parameters	Δρ_max_ = 0.64 e Å^−^^3^
0 restraints	Δρ_min_ = −0.42 e Å^−^^3^

### Special details

**Table:**

Geometry. All esds (except the esd in the dihedral angle between two l.s. planes) are estimated using the full covariance matrix. The cell esds are taken into account individually in the estimation of esds in distances, angles and torsion angles; correlations between esds in cell parameters are only used when they are defined by crystal symmetry. An approximate (isotropic) treatment of cell esds is used for estimating esds involving l.s. planes.
Refinement. Refinement of F^2^ against ALL reflections. The weighted R-factor wR and goodness of fit S are based on F^2^, conventional R-factors R are based on F, with F set to zero for negative F^2^. The threshold expression of F^2^ > 2sigma(F^2^) is used only for calculating R-factors(gt) etc. and is not relevant to the choice of reflections for refinement. R-factors based on F^2^ are statistically about twice as large as those based on F, and R- factors based on ALL data will be even larger.

### Fractional atomic coordinates and isotropic or equivalent isotropic displacement parameters (Å^2^)

**Table:**

	*x*	*y*	*z*	*U*_iso_*/*U*_eq_	
Fe1	0.94793 (3)	0.68146 (3)	0.671097 (18)	0.02226 (10)	
O1	1.03201 (18)	0.41904 (16)	0.64597 (10)	0.0310 (3)	
O2	1.0923 (2)	0.17641 (16)	0.73677 (11)	0.0407 (4)	
O3	0.72359 (19)	0.75376 (18)	0.59390 (11)	0.0375 (4)	
O4	0.50219 (18)	0.69625 (18)	0.57643 (11)	0.0374 (3)	
O5	1.15739 (18)	0.59663 (18)	0.75470 (11)	0.0389 (4)	
O6	1.2694 (2)	0.6171 (2)	0.88633 (14)	0.0562 (5)	
O7	0.93429 (17)	0.92929 (15)	0.62018 (10)	0.0277 (3)	
O8	0.74650 (19)	1.17534 (16)	0.62741 (11)	0.0374 (4)	
O9	1.10045 (19)	0.65543 (17)	0.51796 (10)	0.0381 (4)	
H1W	1.0520	0.6287	0.4794	0.057*	
H2W	1.1441	0.7208	0.4862	0.057*	
O10	0.3395 (3)	0.0526 (3)	0.8643 (2)	0.0869 (8)	
H3W	0.3746	0.1302	0.8621	0.130*	
H4W	0.2631	0.0905	0.8255	0.130*	
O11	0.4508 (2)	0.30309 (19)	0.85471 (13)	0.0492 (4)	
H5W	0.3834	0.3909	0.8701	0.074*	
H6W	0.5154	0.2604	0.8990	0.074*	
O12	0.3125 (2)	0.8118 (2)	1.01257 (12)	0.0526 (4)	
H7W	0.3325	0.8852	0.9728	0.079*	
H8W	0.2998	0.7450	0.9807	0.079*	
N1	0.7780 (2)	0.56023 (18)	0.77262 (11)	0.0252 (3)	
N2	0.8680 (2)	0.82077 (18)	0.81073 (11)	0.0253 (3)	
N3	0.3994 (2)	0.3950 (2)	0.62827 (14)	0.0434 (5)	
H3A	0.4216	0.4829	0.6165	0.065*	
H3B	0.2973	0.4173	0.6275	0.065*	
H3C	0.4290	0.3460	0.6840	0.065*	
H3D	0.4289	0.3600	0.5735	0.065*	
N4	0.2346 (2)	0.0091 (2)	0.56668 (13)	0.0329 (4)	
H4C	0.3112	−0.0730	0.5747	0.049*	
H4D	0.2628	0.0713	0.5216	0.049*	
H4E	0.1520	−0.0156	0.5576	0.049*	
H4F	0.2066	0.0605	0.6236	0.049*	
C1	0.6843 (3)	0.6605 (2)	0.85380 (15)	0.0317 (5)	
H1A	0.6449	0.5941	0.9088	0.038*	
H1B	0.5892	0.7456	0.8276	0.038*	
C2	0.7883 (3)	0.7347 (2)	0.89379 (14)	0.0324 (5)	
H2A	0.7197	0.8098	0.9405	0.039*	
H2B	0.8722	0.6506	0.9306	0.039*	
C3	1.0140 (2)	0.3246 (2)	0.72460 (14)	0.0274 (4)	
C4	0.8884 (3)	0.3989 (2)	0.81065 (14)	0.0298 (4)	
H4A	0.8244	0.3298	0.8379	0.036*	
H4B	0.9449	0.4080	0.8645	0.036*	
C5	0.6256 (2)	0.6764 (2)	0.62043 (14)	0.0277 (4)	
C6	0.6622 (3)	0.5452 (2)	0.70988 (15)	0.0298 (4)	
H6A	0.5600	0.5501	0.7515	0.036*	
H6B	0.7081	0.4406	0.6850	0.036*	
C7	1.1596 (3)	0.6683 (2)	0.82744 (16)	0.0327 (5)	
C8	1.0194 (3)	0.8263 (2)	0.84213 (15)	0.0320 (5)	
H8A	1.0016	0.8440	0.9127	0.038*	
H8B	1.0476	0.9153	0.8021	0.038*	
C9	0.8172 (2)	1.0346 (2)	0.66590 (14)	0.0254 (4)	
C10	0.7585 (3)	0.9832 (2)	0.77436 (14)	0.0284 (4)	
H10A	0.7569	1.0599	0.8178	0.034*	
H10B	0.6472	0.9829	0.7773	0.034*	

### Atomic displacement parameters (Å^2^)

**Table:**

	*U*^11^	*U*^22^	*U*^33^	*U*^12^	*U*^13^	*U*^23^
Fe1	0.02344 (16)	0.01963 (15)	0.02343 (15)	−0.00748 (11)	−0.00147 (10)	−0.00263 (10)
O1	0.0358 (8)	0.0264 (7)	0.0304 (7)	−0.0123 (6)	0.0021 (6)	−0.0037 (6)
O2	0.0507 (10)	0.0224 (7)	0.0425 (8)	−0.0020 (7)	−0.0104 (7)	−0.0049 (6)
O3	0.0385 (9)	0.0395 (8)	0.0382 (8)	−0.0212 (7)	−0.0116 (7)	0.0098 (6)
O4	0.0304 (8)	0.0406 (8)	0.0422 (8)	−0.0095 (7)	−0.0115 (7)	−0.0070 (7)
O5	0.0296 (8)	0.0402 (8)	0.0446 (8)	−0.0003 (7)	−0.0105 (7)	−0.0194 (7)
O6	0.0533 (11)	0.0431 (9)	0.0717 (12)	0.0026 (8)	−0.0379 (10)	−0.0198 (9)
O7	0.0303 (8)	0.0230 (7)	0.0292 (7)	−0.0102 (6)	0.0018 (6)	−0.0036 (5)
O8	0.0390 (9)	0.0231 (7)	0.0429 (8)	−0.0058 (6)	−0.0035 (7)	0.0039 (6)
O9	0.0536 (10)	0.0368 (8)	0.0300 (7)	−0.0254 (7)	0.0042 (7)	−0.0059 (6)
O10	0.0917 (18)	0.0635 (13)	0.121 (2)	−0.0420 (13)	−0.0600 (15)	0.0275 (13)
O11	0.0491 (11)	0.0368 (9)	0.0549 (10)	−0.0059 (8)	−0.0071 (8)	−0.0056 (7)
O12	0.0699 (12)	0.0548 (10)	0.0420 (9)	−0.0291 (9)	−0.0085 (8)	−0.0101 (8)
N1	0.0283 (9)	0.0206 (8)	0.0258 (8)	−0.0087 (7)	−0.0010 (6)	−0.0018 (6)
N2	0.0286 (9)	0.0205 (8)	0.0254 (8)	−0.0071 (7)	−0.0021 (6)	−0.0025 (6)
N3	0.0350 (11)	0.0508 (12)	0.0409 (10)	−0.0070 (9)	−0.0048 (8)	−0.0135 (9)
N4	0.0308 (10)	0.0314 (9)	0.0349 (9)	−0.0094 (8)	−0.0049 (7)	−0.0009 (7)
C1	0.0321 (12)	0.0288 (10)	0.0310 (10)	−0.0111 (9)	0.0084 (8)	−0.0045 (8)
C2	0.0438 (13)	0.0287 (10)	0.0221 (9)	−0.0116 (9)	0.0037 (8)	−0.0046 (8)
C3	0.0308 (11)	0.0236 (9)	0.0312 (10)	−0.0109 (8)	−0.0083 (8)	−0.0044 (8)
C4	0.0371 (12)	0.0226 (9)	0.0276 (9)	−0.0103 (9)	−0.0034 (8)	0.0022 (7)
C5	0.0246 (11)	0.0260 (9)	0.0313 (10)	−0.0059 (8)	−0.0017 (8)	−0.0077 (8)
C6	0.0282 (11)	0.0272 (10)	0.0370 (10)	−0.0138 (9)	−0.0027 (8)	−0.0028 (8)
C7	0.0330 (12)	0.0294 (10)	0.0378 (11)	−0.0095 (9)	−0.0094 (9)	−0.0068 (9)
C8	0.0384 (12)	0.0259 (10)	0.0343 (10)	−0.0102 (9)	−0.0096 (9)	−0.0071 (8)
C9	0.0258 (10)	0.0220 (9)	0.0310 (10)	−0.0108 (8)	−0.0047 (8)	−0.0030 (7)
C10	0.0281 (11)	0.0222 (9)	0.0315 (10)	−0.0047 (8)	−0.0010 (8)	−0.0050 (8)

### Geometric parameters (Å, °)

**Table:**

Fe1—O5	2.1362 (15)	N2—C8	1.474 (3)
Fe1—O7	2.1736 (13)	N2—C2	1.479 (2)
Fe1—O3	2.2036 (15)	N3—H3A	0.8605
Fe1—O1	2.2603 (13)	N3—H3B	0.8446
Fe1—O9	2.2741 (14)	N3—H3C	0.8315
Fe1—N1	2.3015 (16)	N3—H3D	0.8178
Fe1—N2	2.3087 (15)	N4—H4C	0.8003
O1—C3	1.267 (2)	N4—H4D	0.8238
O2—C3	1.249 (2)	N4—H4E	0.8603
O3—C5	1.263 (2)	N4—H4F	0.9157
O4—C5	1.256 (2)	C1—C2	1.509 (3)
O5—C7	1.265 (2)	C1—H1A	0.9700
O6—C7	1.250 (3)	C1—H1B	0.9700
O7—C9	1.272 (2)	C2—H2A	0.9700
O8—C9	1.247 (2)	C2—H2B	0.9700
O9—H1W	0.8318	C3—C4	1.526 (3)
O9—H2W	0.8355	C4—H4A	0.9700
O10—H3W	0.8503	C4—H4B	0.9700
O10—H4W	0.8501	C5—C6	1.519 (3)
O11—H5W	0.8306	C6—H6A	0.9700
O11—H6W	0.8337	C6—H6B	0.9700
O12—H7W	0.8281	C7—C8	1.519 (3)
O12—H8W	0.8377	C8—H8A	0.9700
N1—C6	1.472 (3)	C8—H8B	0.9700
N1—C4	1.476 (2)	C9—C10	1.524 (3)
N1—C1	1.480 (2)	C10—H10A	0.9700
N2—C10	1.473 (2)	C10—H10B	0.9700
			
O5—Fe1—O7	100.92 (6)	H4C—N4—H4F	110.2
O5—Fe1—O3	174.97 (6)	H4D—N4—H4F	105.4
O7—Fe1—O3	83.47 (5)	H4E—N4—H4F	107.6
O5—Fe1—O1	82.60 (5)	N1—C1—C2	111.70 (16)
O7—Fe1—O1	151.04 (5)	N1—C1—H1A	109.3
O3—Fe1—O1	94.76 (5)	C2—C1—H1A	109.3
O5—Fe1—O9	94.18 (6)	N1—C1—H1B	109.3
O7—Fe1—O9	78.64 (5)	C2—C1—H1B	109.3
O3—Fe1—O9	89.08 (6)	H1A—C1—H1B	107.9
O1—Fe1—O9	72.42 (5)	N2—C2—C1	111.31 (16)
O5—Fe1—N1	100.90 (6)	N2—C2—H2A	109.4
O7—Fe1—N1	135.11 (5)	C1—C2—H2A	109.4
O3—Fe1—N1	74.15 (6)	N2—C2—H2B	109.4
O1—Fe1—N1	70.68 (5)	C1—C2—H2B	109.4
O9—Fe1—N1	137.67 (5)	H2A—C2—H2B	108.0
O5—Fe1—N2	74.47 (6)	O2—C3—O1	124.77 (18)
O7—Fe1—N2	72.13 (5)	O2—C3—C4	118.09 (17)
O3—Fe1—N2	104.83 (6)	O1—C3—C4	117.14 (16)
O1—Fe1—N2	135.37 (5)	N1—C4—C3	109.71 (15)
O9—Fe1—N2	145.64 (6)	N1—C4—H4A	109.7
N1—Fe1—N2	76.69 (6)	C3—C4—H4A	109.7
C3—O1—Fe1	114.30 (11)	N1—C4—H4B	109.7
C5—O3—Fe1	119.01 (13)	C3—C4—H4B	109.7
C7—O5—Fe1	120.10 (13)	H4A—C4—H4B	108.2
C9—O7—Fe1	114.48 (11)	O4—C5—O3	124.92 (19)
Fe1—O9—H1W	108.9	O4—C5—C6	117.13 (17)
Fe1—O9—H2W	125.8	O3—C5—C6	117.91 (17)
H1W—O9—H2W	110.1	N1—C6—C5	112.74 (15)
H3W—O10—H4W	103.6	N1—C6—H6A	109.0
H5W—O11—H6W	110.3	C5—C6—H6A	109.0
H7W—O12—H8W	110.5	N1—C6—H6B	109.0
C6—N1—C4	110.25 (15)	C5—C6—H6B	109.0
C6—N1—C1	109.26 (16)	H6A—C6—H6B	107.8
C4—N1—C1	113.59 (15)	O6—C7—O5	123.93 (19)
C6—N1—Fe1	108.49 (11)	O6—C7—C8	119.19 (18)
C4—N1—Fe1	105.15 (12)	O5—C7—C8	116.89 (18)
C1—N1—Fe1	109.94 (11)	N2—C8—C7	109.75 (15)
C10—N2—C8	111.42 (15)	N2—C8—H8A	109.7
C10—N2—C2	113.67 (16)	C7—C8—H8A	109.7
C8—N2—C2	109.64 (16)	N2—C8—H8B	109.7
C10—N2—Fe1	105.16 (11)	C7—C8—H8B	109.7
C8—N2—Fe1	106.44 (11)	H8A—C8—H8B	108.2
C2—N2—Fe1	110.19 (11)	O8—C9—O7	124.40 (17)
H3A—N3—H3B	109.4	O8—C9—C10	118.26 (17)
H3A—N3—H3C	109.9	O7—C9—C10	117.33 (16)
H3B—N3—H3C	110.5	N2—C10—C9	110.49 (15)
H3A—N3—H3D	102.0	N2—C10—H10A	109.6
H3B—N3—H3D	97.3	C9—C10—H10A	109.6
H3C—N3—H3D	126.5	N2—C10—H10B	109.6
H4C—N4—H4D	108.9	C9—C10—H10B	109.6
H4C—N4—H4E	107.9	H10A—C10—H10B	108.1
H4D—N4—H4E	116.7		
			
O5—Fe1—O1—C3	69.29 (14)	O5—Fe1—N2—C8	26.04 (11)
O7—Fe1—O1—C3	168.49 (13)	O7—Fe1—N2—C8	−81.18 (11)
O3—Fe1—O1—C3	−106.39 (14)	O3—Fe1—N2—C8	−159.12 (11)
O9—Fe1—O1—C3	166.08 (15)	O1—Fe1—N2—C8	87.90 (13)
N1—Fe1—O1—C3	−35.04 (13)	O9—Fe1—N2—C8	−48.15 (16)
N2—Fe1—O1—C3	10.34 (17)	N1—Fe1—N2—C8	131.55 (12)
O7—Fe1—O3—C5	−156.62 (15)	O5—Fe1—N2—C2	−92.77 (13)
O1—Fe1—O3—C5	52.44 (15)	O7—Fe1—N2—C2	160.01 (14)
O9—Fe1—O3—C5	124.71 (15)	O3—Fe1—N2—C2	82.07 (13)
N1—Fe1—O3—C5	−15.92 (14)	O1—Fe1—N2—C2	−30.91 (16)
N2—Fe1—O3—C5	−87.09 (15)	O9—Fe1—N2—C2	−166.96 (12)
O7—Fe1—O5—C7	57.10 (17)	N1—Fe1—N2—C2	12.74 (13)
O3—Fe1—O5—C7	−93.4 (6)	C6—N1—C1—C2	−157.54 (16)
O1—Fe1—O5—C7	−152.03 (17)	C4—N1—C1—C2	78.9 (2)
O9—Fe1—O5—C7	136.32 (16)	Fe1—N1—C1—C2	−38.59 (19)
N1—Fe1—O5—C7	−83.43 (17)	C10—N2—C2—C1	80.2 (2)
N2—Fe1—O5—C7	−10.69 (16)	C8—N2—C2—C1	−154.34 (16)
O5—Fe1—O7—C9	−106.42 (13)	Fe1—N2—C2—C1	−37.50 (19)
O3—Fe1—O7—C9	71.08 (13)	N1—C1—C2—N2	52.0 (2)
O1—Fe1—O7—C9	159.12 (13)	Fe1—O1—C3—O2	−157.92 (16)
O9—Fe1—O7—C9	161.46 (14)	Fe1—O1—C3—C4	21.5 (2)
N1—Fe1—O7—C9	11.38 (16)	C6—N1—C4—C3	73.5 (2)
N2—Fe1—O7—C9	−36.83 (13)	C1—N1—C4—C3	−163.54 (16)
O5—Fe1—N1—C6	−155.86 (11)	Fe1—N1—C4—C3	−43.29 (17)
O7—Fe1—N1—C6	86.33 (13)	O2—C3—C4—N1	−164.14 (17)
O3—Fe1—N1—C6	23.24 (11)	O1—C3—C4—N1	16.4 (2)
O1—Fe1—N1—C6	−77.77 (12)	Fe1—O3—C5—O4	−173.21 (14)
O9—Fe1—N1—C6	−47.10 (15)	Fe1—O3—C5—C6	4.5 (2)
N2—Fe1—N1—C6	133.15 (12)	C4—N1—C6—C5	−143.50 (16)
O5—Fe1—N1—C4	−37.92 (12)	C1—N1—C6—C5	91.00 (18)
O7—Fe1—N1—C4	−155.73 (10)	Fe1—N1—C6—C5	−28.85 (18)
O3—Fe1—N1—C4	141.18 (12)	O4—C5—C6—N1	−164.22 (16)
O1—Fe1—N1—C4	40.17 (11)	O3—C5—C6—N1	17.9 (2)
O9—Fe1—N1—C4	70.84 (14)	Fe1—O5—C7—O6	172.42 (18)
N2—Fe1—N1—C4	−108.91 (12)	Fe1—O5—C7—C8	−7.9 (3)
O5—Fe1—N1—C1	84.71 (13)	C10—N2—C8—C7	−151.24 (16)
O7—Fe1—N1—C1	−33.10 (16)	C2—N2—C8—C7	82.04 (19)
O3—Fe1—N1—C1	−96.19 (13)	Fe1—N2—C8—C7	−37.13 (18)
O1—Fe1—N1—C1	162.80 (14)	O6—C7—C8—N2	−148.1 (2)
O9—Fe1—N1—C1	−166.53 (12)	O5—C7—C8—N2	32.2 (3)
N2—Fe1—N1—C1	13.72 (12)	Fe1—O7—C9—O8	−150.62 (16)
O5—Fe1—N2—C10	144.36 (13)	Fe1—O7—C9—C10	28.3 (2)
O7—Fe1—N2—C10	37.14 (11)	C8—N2—C10—C9	79.50 (19)
O3—Fe1—N2—C10	−40.80 (13)	C2—N2—C10—C9	−156.01 (16)
O1—Fe1—N2—C10	−153.78 (11)	Fe1—N2—C10—C9	−35.41 (17)
O9—Fe1—N2—C10	70.17 (16)	O8—C9—C10—N2	−173.41 (17)
N1—Fe1—N2—C10	−110.13 (12)	O7—C9—C10—N2	7.6 (2)

### Hydrogen-bond geometry (Å, °)

**Table:**

*D*—H···*A*	*D*—H	H···*A*	*D*···*A*	*D*—H···*A*
N4—H4F···O2^i^	0.92	1.93	2.821 (2)	166
N4—H4E···O7^ii^	0.86	2.54	2.990 (2)	114
N4—H4E···O7^iii^	0.86	2.15	2.924 (2)	149
N4—H4D···O3^ii^	0.82	2.04	2.844 (2)	167
N4—H4C···O4^iv^	0.80	2.14	2.928 (2)	171
N3—H3D···O4^ii^	0.82	2.11	2.919 (2)	172
N3—H3C···O11	0.83	2.29	3.083 (3)	159
N3—H3B···O9^i^	0.84	2.60	3.184 (3)	127
N3—H3B···O1^i^	0.84	2.29	3.121 (2)	167
N3—H3A···O4	0.86	2.22	3.081 (3)	174
O12—H8W···O6^i^	0.84	1.95	2.783 (2)	170
O12—H7W···O10^v^	0.83	1.92	2.732 (3)	167
O11—H6W···O12^vi^	0.83	1.93	2.749 (2)	169
O11—H5W···O5^i^	0.83	2.66	3.213 (2)	125
O11—H5W···O6^i^	0.83	1.94	2.750 (2)	165
O10—H4W···O2^i^	0.85	1.92	2.767 (3)	179
O10—H3W···O11	0.85	1.88	2.727 (3)	179
O9—H2W···O8^vii^	0.84	1.96	2.753 (2)	159
O9—H1W···O1^viii^	0.83	2.11	2.920 (2)	166

Symmetry codes: (i) *x*−1, *y*, *z*; (ii) −*x*+1, −*y*+1, −*z*+1; (iii) *x*−1, *y*−1, *z*; (iv) *x*, *y*−1, *z*; (v) *x*, *y*+1, *z*; (vi) −*x*+1, −*y*+1, −*z*+2; (vii) −*x*+2, −*y*+2, −*z*+1; (viii) −*x*+2, −*y*+1, −*z*+1.
